# Assessment of nitrogen losses through nitrous oxide from abattoir wastewater-irrigated soils

**DOI:** 10.1007/s11356-016-7438-y

**Published:** 2016-08-24

**Authors:** Raghupathi Matheyarasu, Balaji Seshadri, Nanthi S. Bolan, Ravi Naidu

**Affiliations:** 1 Future Industries Institute, University of South Australia, Mawson Lakes, Adelaide, South Australia 5095 Australia; 2 Global Centre for Environmental Remediation, The University of Newcastle, Callaghan, Adelaide, New South Wales 2308 Australia; 3 Cooperative Research Centre for Contamination Assessment and Remediation of the Environment, P.O. Box 486, Salisbury, Adelaide, South Australia 5106 Australia

**Keywords:** Abattoir, Wastewater, Irrigation, Nitrous oxide, Emission, Greenhouse gas and global warming

## Abstract

The land disposal of waste and wastewater is a major source of N_2_O emission. This is due to the presence of high concentrations of nitrogen (N) and carbon in the waste. Abattoir wastewater contains 186 mg/L of N and 30.4 mg/L of P. The equivalent of 3 kg of abattoir wastewater-irrigated soil was sieved and taken in a 4-L plastic container. Abattoir wastewater was used for irrigating the plants at the rates of 50 and 100 % field capacity (FC). Four crop species were used with no crop serving as a control. Nitrous oxide emission was monitored using a closed chamber technique. The chamber was placed inside the plastic container, and N_2_O emission was measured for 7 days after the planting. A syringe and pre-evacuated vial were used for collecting the gas samples; a fresh and clean syringe was used each time to avoid cross-contamination. The collected gas samples were injected into a gas chromatography device immediately after each sampling to analyse the concentration of N_2_O from different treatments. The overall N_2_O emission was compared for all the crops under two different abattoir wastewater treatment rates (50 and 100 % FC). Under 100 % FC (wastewater irrigation), among the four species grown in the abattoir wastewater-irrigated soil, *Medicago sativa* (23 mg/pot), *Sinapis alba* (21 mg/pot), *Zea mays* (20 mg/pot) and *Helianthus annuus* (20 mg/pot) showed higher N_2_O emission compared to the 50 % treatments—*M. sativa* (17 mg/pot), *S. alba* (17 mg/pot), *Z. mays* (18 mg/pot) and *H. annuus* (18 mg/pot). Similarly, pots with plants have shown 15 % less emission than the pots without plants. Similar trends of N_2_O emission flux were observed between the irrigation period (4-week period) for 50 % FC and 100 % FC. Under the 100 % FC loading rate treatments, the highest N_2_O emission was in the following order: week 1 > week 4 > week 3 > week 2. On the other hand, under the 50 % FC loading rate treatments, the highest N_2_O emission was recorded in the first few weeks and in the following order: week 1 > week 2 > week 3 > week > 4. Since N_2_O is a greenhouse gas with high global warming potential, its emission from wastewater irrigation is likely to impact global climate change. Therefore, it is important to examine the effects of abattoir wastewater irrigation on soil for N_2_O emission potential.

## Introduction

**Fig. 1:**
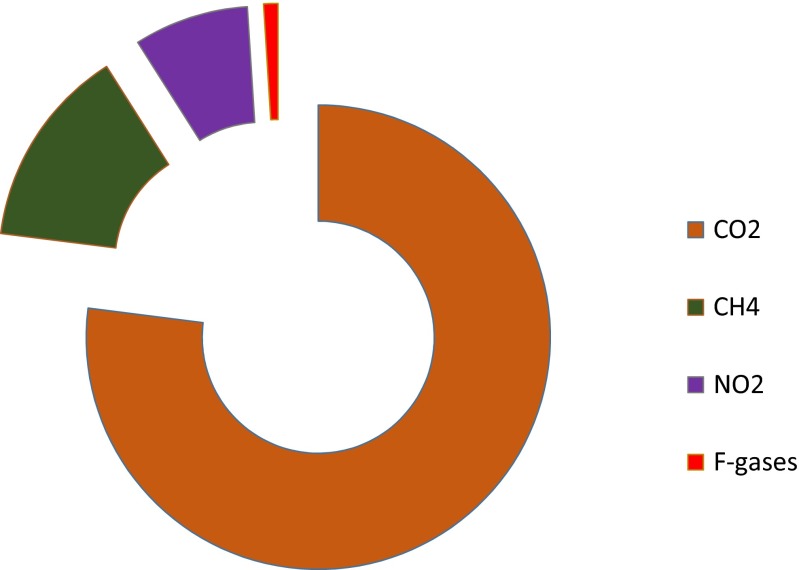
Global greenhouse gases emission (in %) (adapted from IPCC report 2007) (*F-gases* = fluorinated gas (hydrofluorocarbons (HFCs), perfluorocarbons (PFCs), sulphur hexafluoride (SF6) and nitrogen trifluoride (NF3)

The emission of greenhouse gases (GHGs) increases with the rising global population (Preston et al. 2006). Human activities such as agriculture (e.g. chemical fertilisers), energy production (e.g. coal combustion), transport (e.g. fossil fuels) and other industrial activities are directly or indirectly contributing to the GHG emissions (Crutzen et al. 2008). Among the various sources that are responsible for GHG emissions, energy production is the major contributor followed by land use change for agriculture and industrial activities (Meinshausen et al. 2009; Cerri et al. 2009). The major global greenhouse gases and their percentage of emission are illustrated in Fig. 1. At a global scale, nitrous oxide (N_2_O) is a major GHG after carbon dioxide (CO_2_) and methane (CH_4_) (IPCC-2007). However, the global warming potential (GWP) varies between these GHGs, for example, N_2_O is 282 times more powerful than CO_2_ (Ravishankara et al. 2009). Hence, the management of N_2_O by reducing their emission is important in mitigating climate change (McCarl and Schneider 2000). Since N_2_O is a highly potential GHG towards GWP, measurement and mitigation need to be done on a broader scale (Shine et al. 2005). N_2_O emission contributes about 6 % of the overall global warming effect, but its contribution from the agricultural sector is about 16 %. Of that, almost 80 % of N_2_O is emitted from Australian agricultural lands, originating from N fertilisers (32 %), soil disturbance (38 %) and animal waste (30 %) (Dalal et al. 2003).

Globally, denitrification is the primary process of N_2_O production in temperate grassland soils and accounts for 60 % of the total N_2_O emissions (Jha et al. 2012). A recent study at the European Union states that ruminants (cows, sheep and goats) have the highest carbon footprint (FAO 2006). Total net GHG emission of EU livestock production was estimated at 661 mt of carbon dioxide equivalent (CO_2_-eq) which is about 9–13 % of the total GHG emission for the EU agricultural sector, comprising 23 % CH_4_, 24 % N_2_O, 21 % CO_2_ (energy use) and 29 % CO_2_ (land use). A considerable amount of GHGs is emitted by the global animal industry, which is more than all the cars in the world put together, and a large part of that is 18 % N_2_O and CH_4_ emissions; both of these gases have a far more powerful greenhouse gas effect than carbon dioxide (Garnaut et al. 2008).

The livestock sector accounts for 5–50 % of the total contribution, but it may vary from place to place (Gunnarsson et al. 2011). The overall contribution consists of pigs 0.4 %, sheep 3.4 %, cattle 2.7 % and beef cattle 11.2 %, which on average emits 554 kg CO_2_-eq/tonne hot standard carcass weight (MLA 2010). Untreated abattoir wastewater is unsuitable for reuse or discharge into the receiving environment. It will cause serious environmental hazards in the receiving environment such as eutrophication, land degradation, nutrient leaching, groundwater contamination, greenhouse gas emission and effects on ecosystem value; hence, a proper reduction in pollutant levels in the prior stage is essential. In the recent years, meat production and consumption have been increasing considerably and predicted to peak in 2020 predominantly in Asia and the Pacific. Global per capita meat consumption is projected to increase from 32.9 kg/rwt in 2011 to 35.4 kg/rwt in 2020 (OECD-FAO 2011). Meat production is a considerable source of global GHG emission, emitting methane, nitrous oxide and carbon dioxide through various stages. GHGs are emitted by direct energy consumption and indirectly by feedstock production, herding, movement of animals, product transport, slaughtering, cleaning and dressing the animal product, waste and wastewater. The disposal of waste and wastewater to land is a major source of N_2_O emission (Oene et al. 2005; Bolan et al. 2004). N_2_O emission from wastewater-irrigated soils has been explored by many researchers (Bhandral et al. 2007; Saggar et al. 2004; Dalal et al. 2003).

Agricultural industry wastewater such as abattoir and dairy wastewaters are significant contributors towards N_2_O emissions (Kampschreur et al. 2007; Russell et al. 1993). This is due to the presence of the high concentration of N and carbon; for example, abattoir wastewater (AWW) contains 200 to 400 mg/L of N and 545 mg/L of dissolved organic carbon (Longhurst et al. 2000; Cassidy and Belia 2005; Matheyarasu et al. 2012). Tsujimoto (1994) observed that N loss through leaching and gaseous emissions increases with an increasing level of animal waste and wastewater application in soil. Soils are the major source of the greenhouse gas nitrous oxide (N_2_O) in our atmosphere.

The rate of denitrification in soils and the relative proportions of NO, N_2_O and N_2_ produced are controlled by various factors, such as soil microorganisms, climatic factors and management practices. The availability of mineral N (both NH_4_^+^ and NO_3_^−^) and labile C, together with processes that affect reaction rates, such as temperature, pH and redox potential in soil microsites (Saggar et al. 2013). N_2_O can be produced by nitrifiers, denitrifiers and nitrifiers paradoxically denitrifying. Soil moisture conditions are sub-optimal for denitrification, nitrifier denitrification and the major contributor to N_2_O emission (Kool et al. 2011). The majority of studies indicate that a significant amount of N in waste and wastewater is lost as N_2_O emissions (Kampschreur et al. 2009; Czepiel et al. 1995; Bolan et al. 2004; Saggar et al. 2015). Since N_2_O is a potential greenhouse gas with very high GWP, even low emissions can cause serious effects (Shine et al. 2005). In addition, the application of chemical fertilisers alone contributes 46 % of N_2_O emission from the agriculture sector (Baumert et al. 2005) and therefore minimising the usage of these fertilisers will also reduce the emission rate.

There are increased concerns about denitrification associated with the loss of N in the environment. Denitrification can be both detrimental and beneficial to the environment (Bolan et al. 2004). For example, N_2_O, one of the gaseous products from denitrification, has possible deleterious effects on global warming (Bateman and Baggs 2005). The primary consideration for mitigating gaseous N emissions from arable land is to match the supply of mineral N (from fertiliser application, legume-fixed N) to plant needs, although it is possible to achieve uniform application of N fertilisers. Mitigation approaches need to focus on ways to reduce the production of N_2_O during denitrification and enhance the reduction of N_2_O to N_2_ thus lowering the N_2_O:N_2_ product ratio (Saggar et al. 2013). On the other hand, effective management practices help to minimise processes such as leaching, denitrification and NH_3_^−^ volatilisation, all of which lead to the loss of plant-available N from the soil–plant system. These management practices include optimum N supply to pasture crops, proper animal residue management, controlled-release fertiliser and proper water management (Bolan et al. 2004).

Wastewater irrigation increased the concentration of major nutrients (N, P and K) in soil (Matheyarasu et al. 2014). Although there was an increase in soil fertility, the potential for N losses through nitrate leaching and N_2_O emission is likely to be high. There is a great scope and need for reducing N_2_O emissions from various sources to the atmosphere (Ravishankara et al. 2009; McCarl and Schneider 2000). Currently, there are limited reports on N_2_O emission from AWW-irrigated soils or AWW-irrigated cropping system in Australia. Therefore, it is important to examine the effects of AWW irrigation on the GHG emission potential. This paper examines the effect of different types of nutrient source (wastewater and urea) and irrigation intensity on biomass productivity of selected plant species and their N uptake efficiency and N_2_O emission under greenhouse conditions. The overall objective of the study is to examine the effects of AWW irrigation on N uptake and N_2_O emission in a calcareous soil. The specific objectives of the study included (a) to quantify N_2_O emission from agricultural soil treated with various soil moisture conditions and N supplements (urea and AWW), (b) to investigate the effects of AWW irrigation on N_2_O emission and (c) to study the rate of plant-induced denitrification in two different moisture gradients (50 and 100 % FC) with and without plants.

## Materials and methods

### Contaminated site assessment and soil sample collection

The study area (sampling site) is situated at 89.7 km north of Adelaide, South Australia. The latitude and longitude of the study area are 34° 8′ 26.60″ S and 138° 11′ 7.35″ E; the range is 749 m and the elevation of the treatment site is generally flat ranging from 13.5 m Australian height datum (AHD) to 14.5 m AHD. The region has mean annual rainfall of 287.3 mm and annual mean maximum temperature of 22.8 °C and minimum temperature of 10.7 °C. Abattoir wastewater and soil under abattoir wastewater irrigation were collected from land treatment. The AWW-irrigated soil were collected, air-dried and sieved to <2 mm for physiochemical characterisation. The site was under long-term wastewater irrigation to manage wastewater economically and was used for forage production, alternatively. The land treatment site (CI) has received around 385 mm of secondary treated effluent applied over the year at the rate of 32 mm per month.

The CI soil also received an additional 310 mm of water through rainfall, during the period (2012). In the study site, the rate of irrigation was not adjusted according to annual rainfall since it is intended for land treatment. The stored soil samples as collected from different locations and depths were analysed for pH, electrical conductivity (EC), nitrogen (N), phosphorus (P), carbon (C) and micro nutrients. Soil analyses were performed following standard methods as described in the *Soil Chemical Methods—Australasia* (Rayment and Lyons 2011) manual. Soil pH was measured in water using glass electrodes at a 1:5 soil to water ratio. Soil EC was also measured at the same time using an EC meter. Soil total C and total N were estimated by dry combustion on air-dry soil using a LECO 2000 CNS analyser (Sparling et al. 2006). Olsen P was estimated by soil extraction with sodium bicarbonate (0.5 M at pH 8.5) and measured by the molybdenum blue method (Olsen et al. 1954). Absorbance was measured at 882 nm in an Agilent UV–visible spectroscopy system (Germany), and the Olsen P concentration was calculated by preparing a calibration curve against the standards. The total P and micronutrients were determined using inductively coupled plasma-optical emission spectrometry (ICP-OES), with acid-digested soil samples (1:3 ratio of concentrated nitric–hydrochloric acid mixture/aqua regia) (Chen and Ma 2001). Similarly, available N (nitrate-N and ammonia-N) was measured using the SKALAR SANS system (analyser) with potassium chloride (2 M)-extracted soil samples (Luo et al. 2004).

### Plant growth experiment

The plant growth experiment was conducted at the University of South Australia greenhouse using the contaminated soil collected from the land treatment sites. The wastewater used in this experiment was collected from the Primo abattoir at Port Wakefield, which was rich in major plant nutrients such as total nitrogen (TN) and total phosphorus (TP). Two sets of experiments were conducted to examine the effects of wastewater irrigation on N loss through gaseous emission (N_2_O). In experiment 1, urea was used as a N source to study the gaseous emission from the soil. In experiment 2, AWW was used as N source and the effects of adding wastewater at different loading rates towards N_2_O gaseous emission were studied.

### Experiment 1—a study on N_2_O emission without plants in a laboratory condition

Experiment 1 comprised seven treatments with three replicates to study the effects of urea addition on gaseous emission (N_2_O). In this experiment, five different moisture levels were applied (e.g. 25, 50, 75, 100 and 120 % FC) with two levels of nitrogen loading (500 and 1000 mg/kg of soil) to examine the N loss through gaseous emission.

### Experiment 2—gaseous emission with plants

**Plate 1:**
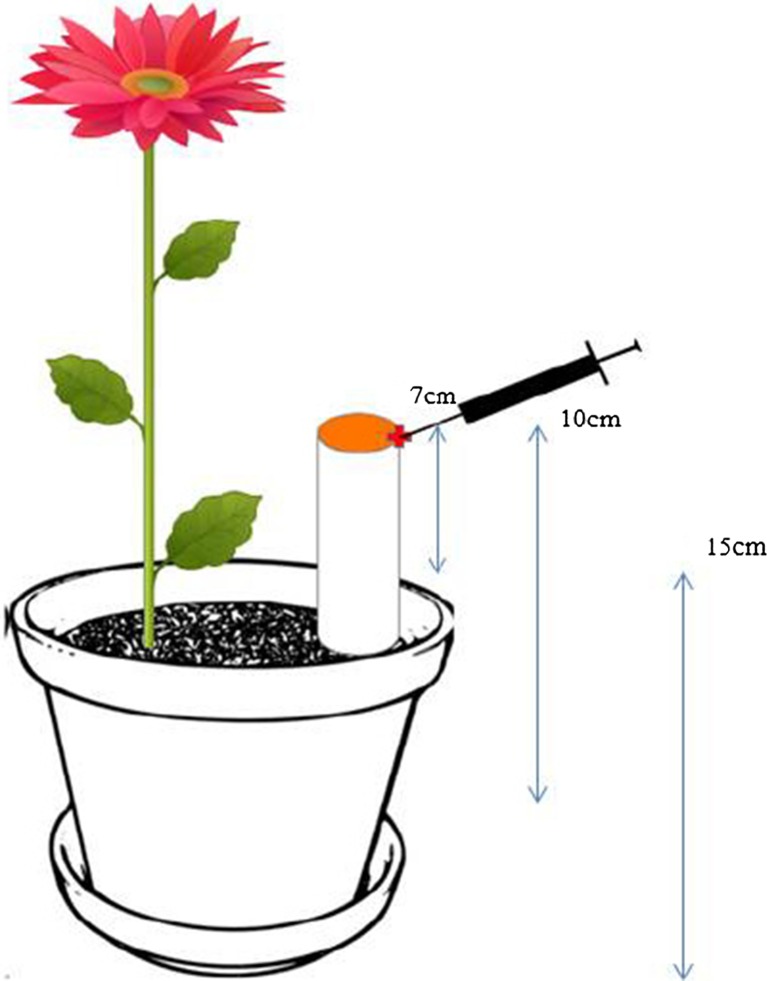
A study on plant-induced denitrification at a greenhouse located at the University of South Australia

The equivalent of 3 kg of soil was sieved and taken in a 4-L plastic container. AWW was used for irrigation of the plants at the rates of 50 and 100 % FC. Crop species including *Helianthus annuus*, *Sinapis alba*, *Medicago sativa* and *Zea mays* were used with no crop serving as a control. The entire experiment was carried out with three replications (Plate 1). Treatment details are as follows: 2 moisture levels*4 + 1 control (no plant)*3 replicates (2*5*3 = 30).

### Gas sampling unit

**Fig. 2:**
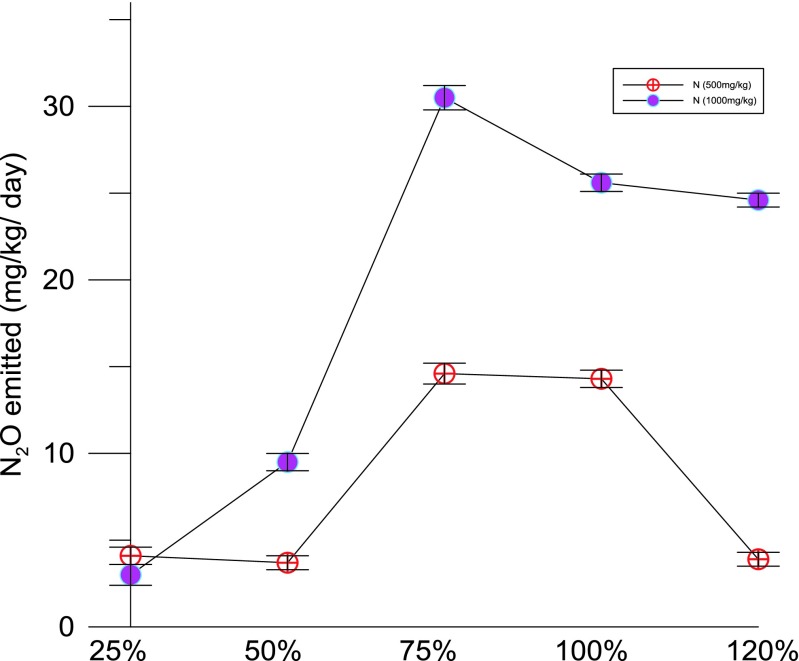
Modified chamber techniques for the measurement of N_2_O emission from the controlled environment

The N_2_O emission from the treatment pot was measured weekly using a modified closed chamber technique. The modified chamber technique was developed during this study based on the principles described by Saggar et al. (2002; 2004) and Bhandral et al. (2007). This modified closed chamber technique involves sampling of N_2_O emission close to the soil surface (10 cm), with the apparatus full description of the modified closed chamber presented in Fig. 2. The closed chamber was placed inside a pot (3 kg of soil). A total of 30 chambers were designed and used in this study with three replicates of each treatment. The dimensions of the chamber were 17 cm in height and 4 cm in diameter. The chamber was inserted into the soil with 7 cm exposed above the soil. Background N_2_O emissions were measured for each pot on the first day after the initial setup to validate and check the efficiency of the modified closed chamber. After the application of treatments, measurements were made every week until 4 weeks after planting.

### Gas sampling

**Plate 2:**
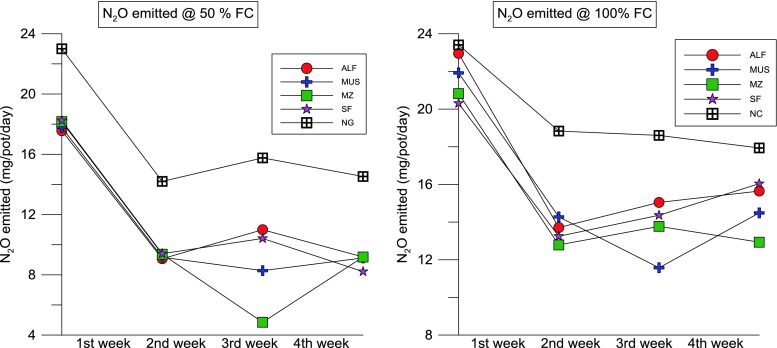
Gas sampling method for N_2_O analysis

Each gas sampling unit was placed inside the pot (Plate 2). Emissions of N_2_O were measured for 7 days after the incubation in experiment 1 and planting in experiment 2 using the active efflux method. A syringe and pre-evacuated vials were used for collecting the gas samples; a fresh and clean syringe was used each time to avoid cross-contamination. The collected gas samples were injected to a gas chromatograph immediately after each sampling to analyse the emission of N_2_O from different treatments. At the end of the greenhouse studies, a nutrient loss percentage as N_2_O (%) was calculated to study the nutrient losses using nutrient input and loss ratio. Plants were harvested after 6 weeks of germination. The shoots and roots were separated and dried in a hot air oven at 70 °C to a constant weight. Plant samples were stored in a dry airtight container for further nutrient analysis to calculate nutrient uptake.

### Statistical analysis

SPSS (Inc., 2001) was used to analyse the data. The differences in the replicates were determined using standard deviations for the gaseous emission (N_2_O). Relationships between wastewater loading types (50 vs 100 % FC) and gaseous emission (N_2_O) were analysed by Pearson correlation coefficients and the two-sample *t* test.

## Results and discussion

### Properties of soils and wastewater used in this experiment

The soils used in this study were collected from an abattoir wastewater-discharged landfill site at Port Wakefield, South Australia. The collected samples were air-dried, characterised for physicochemical properties and used for greenhouse plant growth experiment (assessment of nitrogen losses). Soil collected from the land treatment site was moderately alkaline; the pH of the CI soil was moderately acidic (6.3), with CNI and CTRL measuring 8 and 8.6, respectively. The electrical conductivity was very high ranging from 500 to 1109 μS/cm.

The AWW-irrigated soil was high in TN and TP at available nutrient concentrations. The nutrient contents in AWW-irrigated soil were up to 1165 mg/kg of nitrogen (N) and 223 mg/kg of phosphorus (P). The wastewater sample was characterised for its major nutrient concentration (N and P) immediately after collection. The AWW used in this experiment had a high TN and TP concentration (186 mg/L of N and 30.4 mg/L of P) (Table 1).

**Table 1:** Properties of soil used in these experiments (wastewater-irrigated soil)

Soil parameters	Properties
pH	6.3 ± 1.5
EC (μS/cm)	299.6 ± 1.9
Moisture (%)	11.8 ± 0.1
Total C (mg/kg)	10,523 ± 1.1
Total N (mg/kg)	1165.9 ± 1.4
Nitrate-N (mg/kg)	53.7 ± 0.6
Ammonia-N (mg/kg)	14.9 ± 0.02
Total P (mg/kg)	223.1 ± 0.02
Olsen-P (mg/kg)	73.6 ± 0.08
K (mg/kg)	2849.8 ± 0.01
Ca (mg/kg)	2266.7 ± 0.01
Mg (mg/kg)	709.2 ± 0.09
Na (mg/kg)	308.9 ± 0.05
Fe (mg/kg)	10.4 ± 0.06
Al (mg/kg)	10.7 ± 0.06
B (mg/kg)	4.2 ± 0.02
Zn (mg/kg)	0.4 ± 0.03
Mn (mg/kg)	17.4 ± 0.01

### Effects of urea application and rate of application on nitrous oxide emission with varying soil moisture

The differences in the rate of N application (as urea) on N losses through N_2_O were observed by the end of the experiment. The application of urea with two different rates (low = 500 mg/kg; high = 1000 mg/kg) showed significant impacts on the N_2_O emission. The rate of emission was also significantly affected by the soil moisture content. The results showed that the high rate of N addition (1000 mg/kg) was most vulnerable in terms of N losses with varying moisture gradient. For both the levels of N application, the N_2_O emission increased with the increasing soil moisture content (% FC) up to the saturation (100 % FC) and the rate of N_2_O emission started to decline after the soil reaches the maximum water holding capacity (>100 % FC).

**Fig. 3:**
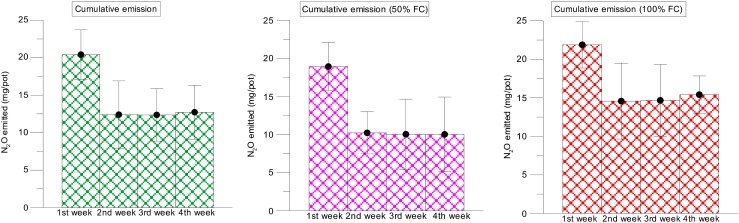
Nitrous oxide emission from soil treated with urea with 500 and 1000 mg/kg—with varying moisture gradient (25, 50, 75, 100 and 120 % FC)

The rate of N_2_O emission will be minimum in a higher FC condition (waterlogged or flooded soils); this is due to less aeration (restricted) and low emission (N_2_O) leading to a complete denitrification process, thereby emitting N_2_ gas (Dalal et al. 2003). The maximum level of N_2_O emission was recorded at 75 to 100 % FC in both N treatment levels (500 and 1000 mg/kg of urea-N). Overall, high N addition with high soil moisture (75 to 100 % FC) resulted in a significant amount of N losses which were about 30 mg/pot/day emitted as N_2_O. This was nearly twofold higher than that of the other treatment level of 500 mg/kg of soil N as urea (Fig. 3). Soil N_2_O production was highly dependent on the oxygen (O_2_) supply and water-filled pore space (WFPS) (Bhandral et al. 2007; Dalal et al. 2003). The high N_2_O emission can be expressed in a situation like low O_2_ partial pressure (<0.5 vol.%) and high WFPS (>60 %) (Saggar et al. 2013). N_2_O is primarily produced in soil by the activities of microorganisms during nitrification and denitrification processes. The ratio of N_2_O production depends on oxygen supply or water-filled pore space, decomposable organic carbon, N and substrate supply (Dalal et al. 2003).

### Effects of AWW irrigation on nitrous oxide emission

#### Effects of nutrient loading

**Fig. 4:**
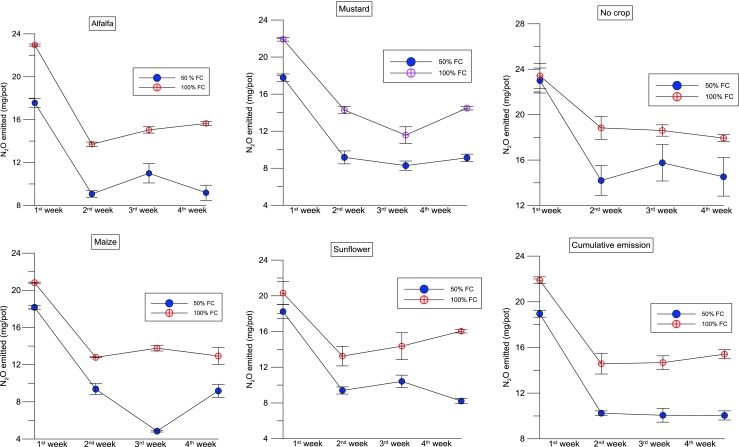
Nitrous oxide emission from soil irrigated with abattoir wastewater with 50 and 100 % FC (moisture gradients)—measured at 4 weeks after planting (*ALF* alfalfa, *MU* mustard, *MZ* maize, *SF* sunflower, *NC* no crop)

Abattoir wastewater is the major source of N to the soil. Irrigation of nutrient-rich AWW increased the soil fertility and nutrient loss, especially N. The soil nutrient loss was significantly higher under AWW irrigation compared to that in non-irrigated soils. The N_2_O emission rate significantly varied between the two levels of wastewater application. The distinct N_2_O peak was observed during the first week after the application of AWW in all the crop species grown and non-cropping condition. The peak was at 22 mg/pot in 100 % FC and 18 mg/pot in 50 % FC in non-cropped conditions (average of the initial period). According to Bowwman (1996), up to 0.16 % of total N applied to the soil can be lost as N_2_O emission within a day after fertiliser application (Dalal et al. 2003; Ruseel 1993). In comparison to all the treatments (including the AWW 50 % FC with and without a crop, AWW 100 % FC with a crop), the AWW treatment without a crop showed statistically significant N_2_O emissions compared to the other treatment levels (Fig. 4). These increases were recorded from the first week after application of AWW. Similar results were found in Bhandral et al. (2007). They observed the peak emission of N_2_O from the meat industry wastewater-irrigated soils in the first few days after treatment.

The peak emission of N_2_O from the AWW 100 % FC (no crop) was 22 % higher than that of the AWW 50 % FC (no crop) which is 53 % times higher than that of the cropped pot. A peak N_2_O emission flux of 21.9 mg/pot/day was recorded under the non-cropping condition at the first week after planting; this indicates that there are greater chances for the loss of applied N if there is no crop to utilise the nutrients. According to Cardenas et al. (2010), high N input can increase the annual N_2_O emission fluxes in a pasture under a range of N fertiliser inputs and their study concluded that higher emissions are possible under increased nutrient supply. A significant increase was recorded in consecutive weeks, but the differences were not much significant as compared to the first week. The overall emission data of 4 weeks suggest that there were highly significant effects of a high load of nutrient addition through wastewater in terms of N loss.

### Effects of time

**Fig. 5:**
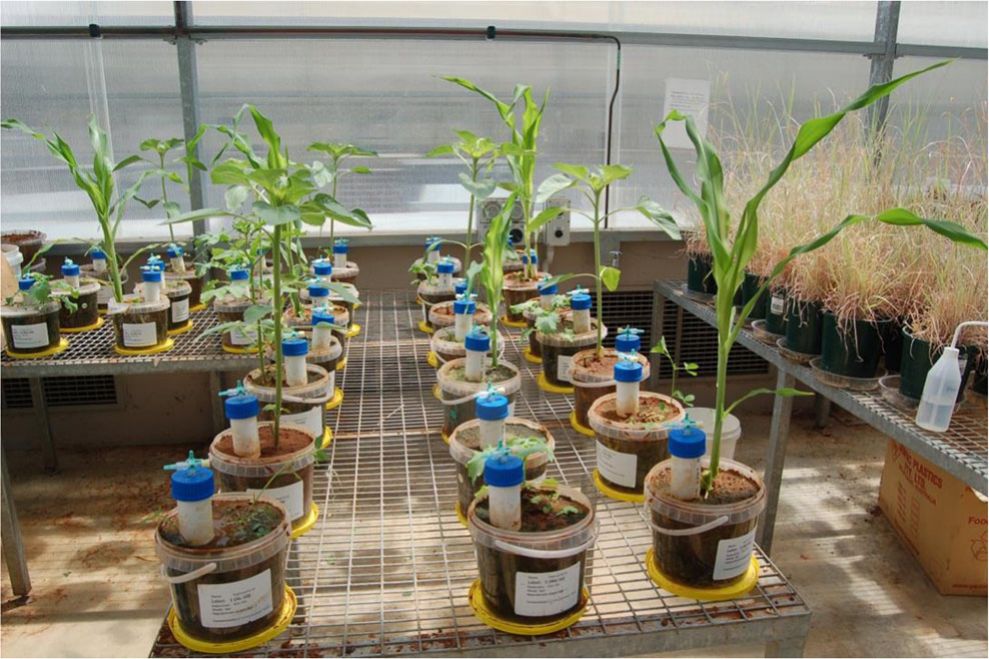
The effects of abattoir wastewater (50 and 100 % FC) on cumulative N_2_O emission of four plant species (alfalfa, mustard, maize and sunflower) measured at the fourth week after planting

The N_2_O emission was significant in 100 % FC at 2 weeks after the plantation in all the pots. The N_2_O emission was high at the first 2 weeks after the planting and declined subsequently to reach the background levels within 4 weeks. Application of AWW irrigation has increased the soil N_2_O emission rate in a short-duration study at the greenhouse for 4 weeks. The total emission in the first week was higher compared to that of the rest of the 3 weeks in both soils. The highest N_2_O emission was recorded in the 100 % FC in the first week for all the four pots, which was about 21 to 22 mg/pot. The AWW irrigation affected N_2_O emissions at both levels of irrigation (50 and 100 % FC). The N_2_O emission increased initially (the first week after the treatment) and declined in the consecutive weeks of the experimental period (for example, the fourth week) (Fig. 5).

Application of AWW (at 100 % FC) increased N_2_O emission by 8 % in the first week and 43 % in the second week after application. A similar study by Singh et al. (2008) states that the application of urine resulted in a high amount of N_2_O losses in their incubation studies. The authors found that the peak of N_2_O emissions was within a week after application of dairy cow urine and observed a maximum of 11.2 mg of N_2_O/kg of soil. Also, their studies showed that the maximum emission was reached before day 25 of the 40-day incubation study (Singh et al. 2008). In the 4-week treatment period, N_2_O emissions from 100 % FC (no crop) treatment in the first 2 weeks remained significantly higher than the N_2_O emitted from the 50 % FC (no crop).

### Effects of plant species used

**Fig. 6:**
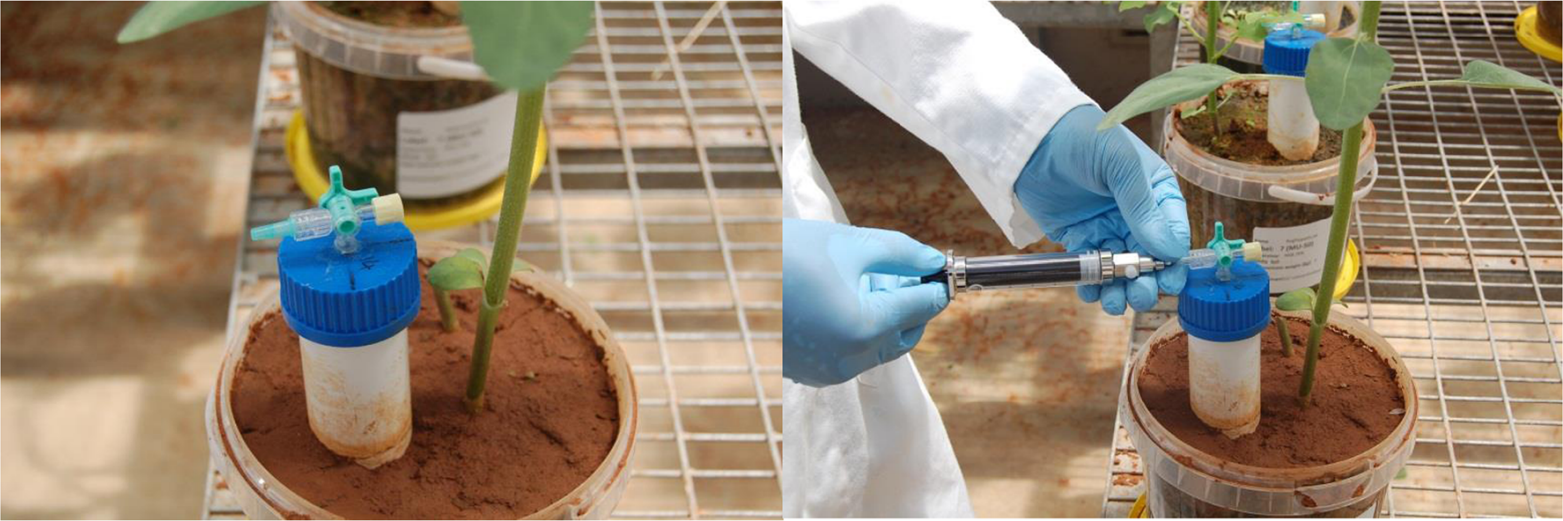
Nitrous oxide emission from soil irrigated with abattoir wastewater with 100 % FC (moisture gradients)—measured at 4 weeks after planting

There was a significant difference between AWW 50 % FC and 100 % FC irrigation in the overall N_2_O emission of all the four crops used in this study. For example, in comparison with 50 % FC, 30, 23, 17 and 11 % (*H. annuus*, *S. alba*, *M. sativa* and *Z. mays*) of increases in N loss were recorded as N_2_O in 100 % FC. N_2_O emission was significantly (*p* < 0.05) higher in the application of 100 % FC of AWW to all the four crops grown. A maximum of 27.5 mg/pot was recorded in the 100 % FC condition in the second week of application with AWW. In general, N_2_O emissions were significantly lower from all the crops irrigated with wastewater than those of the non-crop condition in both levels of treatment (50 and 100 % FC). The cumulative emissions recorded in the 50 % FC were 17.5, 17.7, 18.1 and 18.2 mg/pot for *H. annuus*, *S. alba*, *M. sativa* and *Z. mays*, respectively (non-cropped was 23 mg/pot). Similarly, the cumulative emissions recorded in the 100 % FC were 22, 21, 20 and 20 mg/pot from *H. annuus*, *S. alba*, *M. sativa* and *Z. mays*, respectively (non-cropped was 23.4 mg/pot) (Fig. 6). Pots with plants showed lower emissions than the pots without plants. Overall, the cumulative (average) N_2_O emission was 13 % higher in high-field-moisture pots (100 % FC) than that in the moisture-deficit pots (50 % FC) (Table 2). The second highest N_2_O emission was recorded on the second week after application of the AWW. A similar result was found in Bhandral et al. (2007), and they found that the initial increase in the N_2_O emission after the application of meat industry wastewater declined progressively with time.

**Table 2:** Descriptive statistics: the effects of abattoir wastewater irrigation on N_2_O emission: cumulative effects of 50 and 100 % FC (*n* = 30)

Properties	Range	Minimum	Maximum	Mean	Std. deviation	Variance	Skewness	Kurtosis
Nitrate-N (mg/pot)	621.2	291.4	912.6	489.8	193.1	37,294.2	0.9	–
Ammonia-N (mg/pot)	128.0	60.0	188.0	101.1	39.9	1588.0	0.9	–
N_2_O week 1 (mg/pot)	12.8	14.7	27.5	20.4	3.4	11.4	0.5	−0.5
N_2_O week 2 (mg/pot)	19.9	7.6	27.5	12.4	4.5	20.3	1.8	3.4
N_2_O week 3 (mg/pot)	18.8	3.5	22.3	12.4	5.1	26.2	0.1	−1.0
N_2_O week 4 (mg/pot)	18.6	0.6	19.2	12.7	4.7	21.9	−0.9	0.2
Cumulative emission (mg/pot)	55.8	33.0	88.8	57.9	13.9	193.9	0.1	−0.5

– equal to zero

### Effects of soil moisture

Among the two levels of AWW irrigation treatment, N_2_O emissions were highest for AWW 100 % FC (16.6 mg/pot), whereas the 50 % FC emitted 12.3 mg/pot (cumulative emission average). Bhandral et al. (2007) suggested that the highest N_2_O emission is possible if soil is irrigated/applied with a high dose of urine, ammonium and urea.

The N_2_O emission was higher (66.5 mg/pot) at 100 % FC (*p* < 0.01) than at 50 % FC (49 mg/pot) which may be attributed to higher soil moisture content in the former treatment (Table 3). According to a study by Bateman and Baggs (2005), N_2_O emission was high under the elevated soil moisture condition. Soil N_2_O emission was significantly affected by soil moisture and a nutrient input source (Weier et al. 1993). Similarly, Maag and Vinther (1996) noticed that soil with an optimum moisture condition and high N source can produce increased N_2_O emissions. Results of decreased N_2_O production at low moisture content were obtained by Dalal et al. (2003). A study by Russel et al. (1993) on AWW irrigation to land treatment in a pastureland suggests that, under favourable conditions (e.g. a soil temperature below 12 °C, soil pH 5.9 and abattoir effluent pH of 5), N loss can reach a maximum within a day. Soil moisture was one of the most important factors influencing N_2_O emissions from tropical forest soils (Kiese and Butterbach-Bahl 2002). In moist soils, the reduction of NO_3_^−^ proceeds in a series of steps, producing NO_2_, nitric oxide (NO), nitrous oxide (N_2_O) and N_2_ gas. Denitrification results not only in the loss of a valuable plant nutrient but also in the release of N_2_O (greenhouse gas), which is implicated in the destruction of atmospheric ozone (Bolan et al. 2004). In a flooded soil condition, denitrification has been considered to be a major pathway of N loss (Aulakh et al. 2001). Nitrifier denitrification can be a prime contributor to total N_2_O production from the soil. Nitrifier denitrification contributed more to N_2_O production than total conventional denitrification of NO_3_^−^ at both 50 and 70 % WFPS.

**Table 3:** Effects of abattoir wastewater irrigation on N_2_O emission (*n* = 30); a comparison of cumulative effects of two types of moisture gradient by the two-sample *t* test

Properties	50 % FC	100 % FC	Sig. diff.
N_2_O emitted—week 1 (mg/pot)	19 ± 3.1	21.9 ± 3	n.s.
N_2_O emitted—week 2 (mg/pot)	10.2 ± 2.8	14.6 ± 4.9	*p* < 0.05
N_2_O emitted—week 3 (mg/pot)	10.1 ± 4.6	14.7 ± 4.7	n.s.
N_2_O emitted—week 4 (mg/pot)	10 ± 4.9	15.4 ± 2.4	*p* < 0.001
Cumulative emission (mg/pot)	49.3 ± 12.5	66.5 ± 9.3	*p* < 0.001

*n.s.* not significant

### Statistical significance

The overall emissions of N_2_O were positively correlated with nitrate-N and ammonia-N for all the 4 weeks of treatment, but the N_2_O emission was negatively correlated with plant DM yield. This means that high plant growth can minimise the N loss as N_2_O emission as a result of elevated N uptake. Similar to soil moisture content, N_2_O emission was positively correlated with ammonia-N, pH and Olsen P (Jha et al. 2012). In the current study, the soil was collected from the land treatment site (AWW irrigated) which was rich in soil nutrients such as nitrate-N, ammonia-N and Olsen P. Overall, the plots irrigated with AWW 100 % FC showed significantly higher N loss (N_2_O) than the plots irrigated with 50 % FC for all four plant species used (Table 4). The overall AWW irrigation (50 and 100 % FC) effects on the plant-induced denitrification of four plant species were interpreted using principal component analysis (PCA) for the individual parameters. The effects of the irrigation loading rate on N losses through N_2_O were clearly explained using PCA. The wastewater loading rates (*n* = 30) of both treatments (AWW 100 and 50 % FC) showed distinction from their nutrient loss as N_2_O. The AWW with 100 % FC irrigated pots (all the four plants) greatly varied from the abattoir 50 % FC irrigated sample reflecting the quantity of N loss and their frequency in different time periods. The factor loading float shows clearly the effects of wastewater irrigation on the soil properties.

**Table 4:** Effects of abattoir wastewater irrigation on N_2_O emission (*n* = 30); a comparison of cumulative effects of two types of moisture gradient by Pearson correlation coefficient

Properties	Nitrate-N (mg/pot)	Ammonia-N (mg/pot)	N_2_O week 1 (mg/pot)	N_2_O week 2 (mg/pot)	N_2_O week 3 (mg/pot)	N_2_O week 4 (mg/pot)	Cumulative emission (mg/pot)	DM yield (mg/pot)
Nitrate-N (mg/pot)	1							
Ammonia-N (mg/pot)	0.509**	1						
N_2_O week 1 (mg/pot)	0.146	0.017	1					
N_2_O week 2 (mg/pot)	0.348	0.035	0.655**	1				
N_2_O week 3 (mg/pot)	0.252	0.159	0.432*	0.255	1			
N_2_O week 4 (mg/pot)	0.282	0.336	0.570**	0.620**	0.488**	1		
Cumulative emission (mg/pot)	0.335	0.187	0.804**	0.784**	0.719**	0.854**	1	
DM yield (mg/pot)	−0.076	−0.246	−0.391*	−0.316	−0.324	-0.285	−0.412*	1

*Correlation is significant at the 0.05 level; **correlation is significant at the 0.01 level

Similar to a high loading rate, factors 1 (nitrate-N) and 2 (ammonia-N) explained 51 and 21 % of variation, respectively. Therefore, nearly 50 % of the total N loss as determined by the nutrient load was supplied through wastewater addition. The properties such as ammonia-N and nitrate-N were largely influenced (factors 1 and 2) by the overall emission of N_2_O. The PCA method showed that AWW irrigation was significantly different in the individual treatments in terms of soil N loss and DM yield in the current pot experiment. The highest N_2_O emission was recorded at 100 % FC, due to the supply of a high rate of nitrate-N through AWW.

### Effects of AWW irrigation on plant-induced denitrification—nutrient loss and utilisation

This study demonstrated the influence of AWW irrigation on the plant-induced denitrification and soil productivity. The effects of AWW irrigation on soil nutrient loss as N_2_O are presented in Table 5. In the present study, the nutrient input included wastewater- and soil-derived nutrients; the output included plant uptake and loss through N_2_O emission. Using the above information, the percentage of N loss through N_2_O was calculated to study the effects of AWW irrigation on plant-induced denitrification, and soil fertility and productivity changes (Eq. 1).

**Table 5:** Effects of abattoir wastewater irrigation on N loss as N_2_O emission (%) in two types of loading rate in four crops grown under greenhouse condition (*n* = 30)

Crops	N added through effluent (mg/pot)		N emitted (mg/pot)		% of N emitted		DM yield (mg/pot)	
	50 % FC	100 % FC	50 % FC	100 % FC	50 % FC	100 % FC	50 % FC	100 % FC
Alfalfa	973.2	2031.9	17.6	23.0	1.8	1.1	7333.3	9333.3
Mustard	973.2	2031.9	17.8	21.9	1.8	1.1	14,333.3	16,666.7
Maize	973.2	2031.9	18.2	20.8	1.9	1.0	24,666.7	32,666.7
Sunflower	973.2	2031.9	18.2	20.3	1.9	1.0	28,666.7	39,333.3
No crop	973.2	2031.9	23.0	23.4	2.4	1.2	0	0

1 $$ \%\mathrm{of}\ \mathrm{added}\ \mathrm{N}\ \mathrm{emitted}=\frac{\mathrm{N}\ \mathrm{emitted}\ \left(\mathrm{mg}/\mathrm{pot}\right)}{\mathrm{N}\ \mathrm{added}\ by\ AWW\ \left(\mathrm{mg}/\mathrm{pot}\right)}\times 100 $$

The results from this study showed that 50 % FC treated soil had higher N_2_O emissions in terms of percentage losses (high differences in input and nutrient loss ratio) compared to the 100 % FC. Similarly, the non-cropped soils were very highly variable between the crops due to the absence of plants. In the current experiment, results showed that nearly 1.2 to 2.4 % of applied N was lost as N_2_O. Moiser et al. (1996) showed that nearly 2.5 % of the total N applied to the soil was lost as N_2_O from agriculture land. The relationship between N input and overall N_2_O emission found in this study was positively correlated, similar to the previous research report by IPCC (1999) and studies by Dobbie et al. (1999).

## Discussion

The result from the greenhouse experiments clearly demonstrated that the addition of nutrients or AWW irrigation enhanced the nitrous oxide emission. The effect of nutrient (N) addition on the soil generally increases the soil N loss through N_2_ and N_2_O, if it is not utilised by green cover, and the excess N may find a pathway to enter the atmosphere or be leached to groundwater (Reichenau et al. 2016). The following reasons could be attributed to the high nitrous oxide emission from the wastewater-irrigated soils: (i) the rate of nutrient loading through abattoir wastewater irrigation/high soil fertility, (ii) the process of denitrification and (iii) the anaerobic condition created by the excessive addition of abattoir wastewater. The rates of nutrient addition on nitrous oxide emission in soil have been reported by many researchers; for example, Zhang et al. (2016) reported that increased nitrate or phosphate loading resulted in stronger influence on denitrification than single nutrient additions that stimulate denitrification rates in sandy sediments in aquatic/terrestrial transition zones.

The abattoir wastewater irrigation had significant impacts on the physiochemical properties of the soils. Nitrous oxide (N_2_O) emissions can be significantly affected by the amounts of N available in soils, and also forms of nutrients, local climate and soil conditions are the determining factors of the effects (Peng et al. 2011). Increased N availability generally leads to increased N_2_O emission (Davidson et al. 1996) because this stimulates the microbial processes of nitrification and denitrification, which produce the gas fluxes. The relationship between N input (500 and 1000 mg/kg) on cumulative N_2_O emission found in this study was significant (N losses increase with the increasing application of N) (Fig. 3). The results have been consistent with earlier reported studies on N_2_O emissions (Liu et al. 2005; van Groenigen et al. 2004; Willén et al. 2016) (Fig. 3). Similarly, Chmura et al. (2016) assessed the impact of nutrient additions on greenhouse gas fluxes using dark static chambers in a microtidal and a macrotidal marsh (Both were experimentally fertilised for 6 years). They found that N_2_O fluxes are likely to vary with the source of pollutant nutrients, but emissions will be lower if N is not accompanied by an adequate supply of P.

Overall, the application of AWW caused significant N_2_O loss. In our experiment, we compared the two treatments of nutrient loadings 50 % FC and 100 % FC and we found that there was a highly significant correlation between both treatments in terms of nitrous oxide emission (*p* < 0.001) (Fig. 4). A number of studies have reported the effect of split fertilisation or low-rate application which showed significant reduction in N_2_O emissions (Yu et al. 2016). There was a higher percentage reduction (28 %) in cumulative N_2_O emissions under the split urea application compared with the single fertilisation, although these emissions were influenced by the N fertiliser rate and soil moisture (Yu et al. 2016). The results suggest that low-rate application of AWW is a potential strategy for reducing N_2_O emissions in a wastewater-irrigated soil or in a land treatment site. Similarly, the results by Neto et al. (2016) showed that N fertiliser increased N_2_O emissions from the soil, especially when urea was used. The emission factor for N fertiliser was 0.46 ± 0.33 %.

There was no difference between urea fertiliser and abattoir wastewater nutrient addition on nitrous oxide emission values. Values of N_2_O fluxes were reported for each week and compared with cumulative emission. The application of AWW at the rate of 100 % FC reached a peak of 24 mg/pot emission in the first few weeks after planting (Fig. 5). A study by van der Weerden et al. (2016) recommended an N_2_O emission inventory for New Zealand’s agricultural soils and found that there was no difference between urea fertiliser in terms of N_2_O emission due to the different origins and characteristics of these N sources. For example, in New Zealand’s agricultural soils, N_2_O emissions have means of 0.6 and 0.3 % for urea fertiliser and FDE, respectively (der Weerden et al. 2016).

According to a study report by Rowe et al. (2012), the effect of N deposition on mineralisable N stock was more apparent in more organic soils, whereas the effect on nitrate proportion was more apparent in more mineral soils. With the high proportions of nitrate (over 40 %) that responses also depend on soil C content and site temperature (Table 1). The proportions of mineral N and nitrate were both strongly influenced by the N deposition rate and by interactions with soil C content (Rowe et al. 2012). In soil, urea is rapidly hydrolysed to ammonium (NH_4_^+^) ions, a part of which may be lost as ammonia (NH_3_^−^) and subsequently as nitrous oxide (N_2_O) (Singh et al. 2013). The rate of denitrification was higher in soils incubated at saturation than in soils incubated at FC. Brown et al. (2012) found that denitrification was the dominant microbial source of N_2_O, and responded to increased soil water content and higher labile carbon availability. Elevated precipitation increased soil emissions of N_2_O, especially in combination with added nitrogen and heat. The reduction to N_2_ plus absorption by water primarily depends on soil properties, such as the availability of mineral N (substrate for nitrification and denitrification), soil oxygen and water content, soil temperature, pH and redox conditions, and the availability of labile organic C and N (Chapuis et al. 2007; Yan et al. 2012). Urease inhibitor increased the plant N uptake but did not result in a significant increase in herbage DM yields from urea fertiliser. Urease inhibitor was effective in reducing NH_3_^−^ and N_2_O emissions from both the urine and urea treatments, with the reduction in N_2_O emissions varying with plant N uptake (Singh et al. 2013). However, the effect of changing soil moisture on DR and N_2_O/N_2_ ratio may vary with the type of soil, its nutrient status and the management practices followed on the farm (Jha et al. 2012).

At wastewater irrigation sites, nitrate is formed during the irrigation events. The soil air is rapidly displaced by the wastewater and the soil becomes saturated. Denitrification and nitrous oxide emission rates are at a maximum during this period. As the site drains, the number of anoxic sites decreases and background rates re-establish (Russell et al. 1993). Peng et al. (2011) observed that peak N_2_O fluxes induced by N treatments were concentrated in short periods (2 to 3 weeks) after fertilisation in summer and in soil thawing periods in early spring. The weekly N_2_O emission was calculated for each crop, and we found significant differences among the crops used with two levels of AWW irrigation. Overall, the N_2_O fluxes were significantly higher in 100 % FC compared to 50 % FC (Fig. 6) probably due to a larger amount of AWW irrigation with nutrients (Table 1). The three N levels increased annual N_2_O emissions significantly (*P* < 0.05) in the medium and high N loading treatments compared with the control. A similar pattern was reported by Russell et al. (1993) in which peak rates at the pasture sites were higher with primary-treated effluent (1–137 g N_2_O-N ha/h) than with anaerobic effluent (1–62 g N_2_O-N ha/h). This was attributed to the higher organic carbon concentration in primary-treated effluent and possibly soil temperature peak nitrous oxide emission rates increased with increasing surface soil temperature. A study by Bhandral et al. (2007) found that among the N sources, the highest emissions were measured with nitrate application, emissions being ten times more than those from other N sources for compacted soil. Also, they reported that the soil compaction caused a sevenfold increase in the N_2_O flux; the total N_2_O fluxes for the entire experimental period ranged from 2.62 to 61.74 kg N_2_O-N/ha for the compacted soil and 1.12 to 4.37 kg N_2_O-N/ha for the uncompacted soil.

The N_2_O emissions were very low in pots with high DM yield as found in this study and shown in Table 5, possibly due to a high amount of nutrient uptake by the crops for growth and development. On the other hand, not-cropped conditions show a higher nutrient loss (high N_2_O emission fluxes recorded in this study) (Table 5). The increased N_2_O emission from the control pot was due to the absence of crop or nutrient utilisation by crops. A twofold increase in the N_2_O emission rate was recorded for the non-cropped pot compared to the cropped pot. In this short-duration study, major peaks were observed on the first 2 weeks after the treatment including the cropped and non-cropped control; this might have continued to increase for a few weeks after the treatment if we continued to irrigate the field at the same irrigation rate (Bhandral et al. 2007). Soils are the main sources of the greenhouse gas N_2_O. The N_2_O emission at the soil surface is the result of production and consumption processes of agricultural systems (Chapuis et al. 2007).

In grazed pastures, loss of N occurs mainly through ammonia (NH_3_^−^) volatilisation, the release of gaseous N such as nitric oxide (NO) and nitrous oxide (N_2_O) through biological denitrification, and nitrate (NO_3_^−^) leaching, which has both economic and environmental implications (Bolan et al. 2004). In the nearly water-saturated soil (90 % WFPS), N_2_O production was, as expected, dominated by conventional denitrification of NO_3_^−^ (Kool et al. 2011). A similar result was reported by Weitz et al. (2001) and Zhou et al. (2008) that N_2_O emission was insensitive to soil moisture, possibly resulting from lower soil nitrogen content (0.13–0.21 %). Differences were observed in nitrification–denitrification rates in flooding conditions because they control the availability of N and oxygen. Nitrification was controlled not only by oxygen but also by the amount of ammonium sulphate added and the high pH of the water; these two latter factors could increase the NH_3_ concentration, with inhibition of the coupled nitrification–denitrification (Carrasco et al. 2004). The results of Bateman and Baggs (2005) indicate N_2_O production during heterotrophic nitrification in our soil at 50 % WFPS and the possibility of aerobic denitrification. Thus, several processes may simultaneously produce N_2_O in soil at 60 % WFPS and below. Nitrification was the main source of N_2_O in soils at 35–60 % WFPS, indicating the significance of this process for global warming.

## Conclusions

A nutrient-rich water source (e.g. AWW) can supply sufficient or a surplus amount of primary nutrients to the soil and plants. However, a significant amount of applied nutrient (N) is lost to air, soil and water through various processes by N_2_O and ammonia (NH_3_) emissions and nitrate leaching. These losses are considered environmental hazards due to the ill effects caused by N_2_O as a potential greenhouse gas and nitrate in groundwater as a potential water pollutant. Hence, minimising and management of nitrogenous pollutants become more important in the current era of sustainable agriculture. The mitigation options to reduce N loss include the use of nitrogenous inhibitors (NI), growing bioenergy crops and adopting efficient farm budgeting (applying nutrients only when it is necessary). The emission of nitrous oxide was high at the first 2 weeks after planting and declined subsequently reaching the background levels within 4 weeks. Nitrous oxide emission was higher for 100 % FC than for 50 % FC. The peak nitrous oxide emission flux was recorded in the non-cropping condition during the first week after planting; this indicated that there are higher risks of applied nitrogen loss through nitrous oxide emission in the absence of plant uptake of nitrogen. Overall, nitrous oxide emission was 15 % higher in the non-cropped than cropped treatments. The conclusions that can be drawn from this study in relation to plant-induced denitrification are as follows: the N_2_O emission rate increased with increasing soil moisture content (50 to 100 % FC), the N_2_O emission rate increased with increasing N input through chemical fertiliser or wastewater irrigation, the N_2_O emission rate decreased in the presence of plants and increased under the no-crop condition due to plant uptake and dry matter yield was found to be significantly higher in the 100 % FC than the 50 % treatment. The rate of N loss varied with the rate of AWW irrigation; nearly 1.2 to 2.4 % of applied N was lost as N_2_O. The main conclusions that can be drawn from this study in relation to plant-induced denitrification are the following: the N_2_O emission rate increased with increasing soil moisture content, the N_2_O emission rate increased with increasing N input through wastewater irrigation and the N_2_O emission rate decreased with the presence of plants which can be attributed to plant uptake.
